# FGF‐2 suppresses expression of nephronectin via JNK and PI3K pathways

**DOI:** 10.1002/2211-5463.12421

**Published:** 2018-04-19

**Authors:** Tadashi Kato, Atsushi Yamada, Mikiko Ikehata, Yuko Yoshida, Kiyohito Sasa, Naoko Morimura, Akiko Sakashita, Takehiko Iijima, Daichi Chikazu, Hiroaki Ogata, Ryutaro Kamijo

**Affiliations:** ^1^ Department of Biochemistry School of Dentistry Showa University Tokyo Japan; ^2^ Department of Internal Medicine Showa University Yokohama Northern Hospital Japan; ^3^ Department of Oral and Maxillofacial Surgery Tokyo Medical University Japan; ^4^ Department of Perioperative Medicine Division of Anesthesiology School of Dentistry Showa University Tokyo Japan; ^5^ Department of Integrative Physiology Shiga University of Medical Science Japan

**Keywords:** fibroblast growth factor‐2, Jun N‐terminal kinase, MC3T3‐E1, nephronectin, phosphoinositide‐3 kinase

## Abstract

Nephronectin (Npnt), an extracellular matrix protein, is a ligand for integrin α8β1 and is involved in the development of various organs, such as the kidneys, bones, liver, and muscles. Previously, we found that Npnt expression was inhibited by various cytokines including transforming growth factor‐β (Tgf‐β) and oncostatin M (Osm). Fibroblast growth factor (Fgf)‐2, otherwise known as basic Fgf, also plays important roles in skeletal development and postnatal osteogenesis. In this study, Npnt expression was found to be suppressed by Fgf‐2 in MC3T3‐E1 cells, an osteoblast‐like cell line, in a dose‐ and time‐dependent manners. Furthermore, Fgf‐2‐mediated *Npnt* mRNA suppression was shown to involve the Jun N‐terminal kinase (JNK) and phosphoinositide‐3 kinase (PI3K) pathways. Together, our results suggest that FGF‐2 suppresses *Npnt* gene expression via JNK and PI3K pathways.

**Abbreviations**
Fgf: fibroblast growth factor
JNK: Jun N‐terminal kinase
MAPK: mitogen‐activated protein kinase
MEMα: alpha‐minimum essential medium
Npnt: nephronectin
Osm: oncostatin M
PI3K: phosphoinositide‐3 kinase
Tgf‐β: transforming growth factor‐β

Nephronectin (Npnt), identified in osteoblast‐like MC3T3‐E1 cells, is an extracellular protein with an epidermal growth factor‐like repetitive structure. Moreover, Npnt is the primary ligand for α8β1 integrin and plays an important role in regulation of cell adhesion, differentiation, and spreading, as well as survival of various organs, such as the kidneys, bones, liver, and muscles 1. *In vivo*,* Npnt* expression is particularly prominent at epithelial–mesenchymal interfaces in tissues undergoing morphogenesis 2, 3. Therefore, elucidation of how the *Npnt* gene is expressed is important for understanding the association between bone development and cell adhesion 4, 5.

MC3T3‐E1 is a cloned mouse osteoblast‐like cell line that retains the synthetic functions of bone and has been utilized as an *in vitro* bone model of development systems. Using MC3T3‐E1 cells, Kahai *et al*. 4 showed that some endogenous miRNAs might repress *Npnt* expression, resulting in a lower level of osteoblast differentiation. Other studies have also reported that transforming growth factor‐β (Tgf‐β) and oncostatin M (Osm) downregulate *Npnt* expression in both dose‐ and time‐dependent manners, while osteoblast differentiation induced by *Npnt* was found to be inhibited by Tgf‐β and Osm in MC3T3‐E1 cells 6, 7.

Results of phylogenetic analysis suggested that 22 different *Fgf* genes can be arranged into seven subfamilies containing 2–4 members each 8. In another study, various growth factors, including fibroblast growth factor‐2 (Fgf‐2), transforming growth factor‐? (Tgf‐β), insulin‐like growth factor‐1 (Igf‐1), platelet‐derived growth factor, and prostaglandin E_2_, were shown to act as autocrine and paracrine hormones for regulation of bone cell proliferation 9. Fgf‐2, which is stored in the extracellular matrix and expressed in osteoblasts 10, influences proliferation and differentiation of a variety of cell types *in vitro* 11, 12. In bone cell culture experiments, Fgf‐2 showed increased replication and reduced differentiation markers, such as alkaline phosphatase and type I collagen 13, 14, 15, thus is considered to have important functions in bone homeostasis.

This study was designed to examine the effects of Fgf‐2 on expression of *Npnt* and related molecular mechanisms. Our results revealed that *Npnt* expression in MC3T3‐E1 cells is regulated by Fgf‐2 via the Jun N‐terminal kinase (JNK) and phosphoinositide‐3 kinase (PI3K) pathways.

## Materials and methods

### Cell culture

MC3T3‐E1 cells were maintained in alpha‐minimum essential medium (MEMα) with 2 mm l‐glutamine and 10 mg·L^−1^ phenol red medium (Cat. No. 135‐15175; Wako Pure Chemical Industries, Ltd., Osaka, Japan), supplemented with 10% FBS (Cat. No. FB‐1365/500; Biosera, Rue de la Calle, France) and 1% penicillin–streptomycin (Cat. No. 15240062; Gibco, Waltham, MA, USA) at 37 °C in a CO_2_ incubator (5% CO_2_, 95% air). For the experiments, cells were plated at 1.0 × 10^5^ in 6‐well plates (Cat. No. 140675; Thermo Scientific Inc., Waltham, MA, USA).

### Reagents

Recombinant murine Fgf‐1, Fgf‐2, Fgf‐8b, Fgf‐9, and Fgf‐23 were purchased from Peprotech (Rocky Hill, CT, USA). PD98059 (Cat. No. P215‐1 mg), SB203580 (Cat. No. S8307‐1 mg), and SP600125 (Cat. No. S5567‐10 mg) were from Sigma (St. Louis, MO, USA), LY294002 (Cat. No. 440202‐5 mg) was from Calbiochem (Darmstadt, Deutshland), and BGJ398 (Cat. No. 872511‐34‐7‐5 mg) was from Selleckchem (Houston, TX, USA).

### RT–PCR

Total RNA was extracted using TRIzol reagent (Cat. No. 15596018; Life Technologies, Carlsbad, CA, USA) according to the manufacturer's instructions. We synthesized cDNA in a reaction mixture containing RNA using SuperScript III (Cat. No. 18080‐044; Life Technologies) and random hexamer (Cat. No. N8080127; Invitrogen, Carlsbad, CA, USA) and then performed incubation at 50 °C for 60 min, followed by inactivation of the reaction by heating at 70 °C for 15 min. PCR was performed with Taq polymerase (Cat. No. M7123; Promega, Madison, WI, USA) using the following specific PCR primers: glyceraldehyde 3‐phosphate dehydrogenase (*Gapdh*), 5′‐GAAGGTCGGTGTGAACGGATTTGGC‐3′, and 5′‐CATGTAGGCCATGAGGTCCACCAC‐3′; *Fgfr1*, 5′‐TGGAGTTCATGTGTAAGGTG‐3′ and 5′‐ATAAAGAGGACCATCCTGTG‐3′; *Fgfr2*, 5′‐AAATACCAAATCTCCCAACC‐3′ and 5′‐GCCGCTTCTCCATCTTCT‐3′; *Fgfr3*, 5′‐ACTGTACTCAAGACTGCAGG‐3′ and 5′‐GTCCTTGTCAGTCGCATCAT‐3′; and *Fgfr4*, 5′‐TACAGTGGCTGAAACACGTCGTCA‐3′ and 5′‐ACAAGCAGAACCAGTGAGCCTGAT‐3′. A 2 μL cDNA sample was used in a 10 μL reaction solution containing Red Taqr® PCR mix (Cat. No. R2523‐20RXN; Sigma). Primers were amplified using a program starting with 1 min of denaturation at 94 °C, followed by 30 cycles of 30 s of denaturation at 94 °C, 30 s of annealing at 58 °C, and 30 s of extension at 74 °C, with a final extension of 1 min at 74 °C.

Real‐time PCR was performed using a StepOne™ Real‐time PCR System (Applied Biosystems, Waltham, MA, USA) with SYBR Green Fast PCR Master Mix (Applied Biosystems) with the following specific PCR primers: *Gapdh*, 5′‐AAATGGTGAAGGTCGGTGTG‐3′ and 5′‐TGAAGGGGTCGTTGATGG‐3′; and *Npnt*, 5′‐CACGAGTAATTACGGTTGACAACAG‐3′ and 5′‐CTGCCGTGGAATGAACACAT‐3′. The total reaction volume was 10 μL including 2 μL of a cDNA sample. The thermos‐cycling parameters employed were holding for 20 s at 95 °C, followed by 40 cycles of denaturation at 95 °C for 1 min, and annealing and extension at 60 °C for 20 s. Amplified products were determined using a standard curve analysis, and the expression level of each gene was normalized against that of *Gapdh* and expressed as the relative value for each experiment.

### Western blotting

Cell lysates were collected using sample buffer solution with reducing reagent (6×) for SDS/PAGE (Cat. No. 09499‐14; Nacalai Tesque, Kyoto, Japan), then electrophoresed onto a 10% SDS/PAGE, and blotted onto a poly(vinylidene difluoride) membrane. The membranes were incubated with anti‐nephronectin (Cat. No. AF4298; R&D Systems, Minneapolis, MN, USA) and anti‐actin (Cat. No. A5060; MERCK, Darmstadt, Deutchland) as the first antibodies and then further probed with anti‐mouse IgG horseradish peroxidase‐linked (Cat. No. NA931V; GE Healthcare, Little Chalfont, UK) and anti‐goat IgG horseradish peroxidase‐linked (Cat. No. NB7352; NOVUS, Littleton, CO, USA) secondary antibodies. Proteins were visualized using ECL Prime Western Blotting Detection reagent (Cat. No. #RPN2232; GE Healthcare).

### Statistical analysis

**Figure 1:**
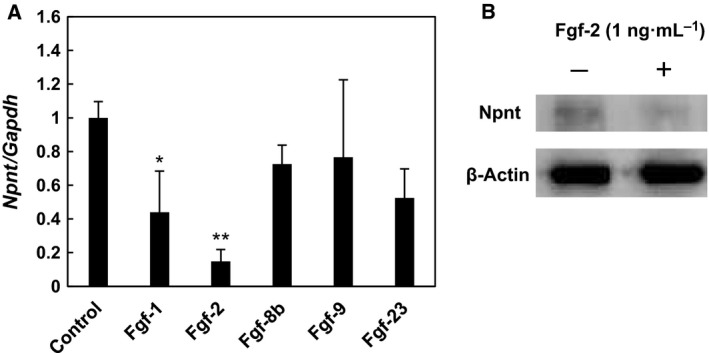
Downregulation of *Npnt* mRNA expression by Fgfs. (A) Real‐time PCR analysis was performed using cDNA from MC3T3‐E1 cells treated with Fgf‐1, Fgf‐2, Fgf‐8b, Fgf‐9, or Fgf‐23 (1 ng·mL^−1^) for 24 h. Values are shown as the mean ± SD of 3 samples as compared to without Fgf treatment. *P < 0.05, **P < 0.01; relative to level in cells without treatment (ANOVA). (B) Western blotting analysis of *Npnt* protein levels in cells treated with or without 1 ng·mL^−1^ of Fgf‐2. Cell lysates were collected after 24 h of incubation.

**Figure 2:**
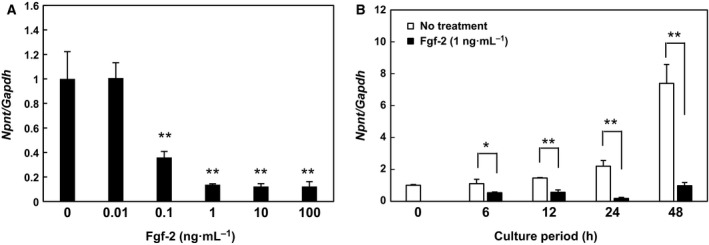
Inhibitory effects of Fgf‐2 occur in dose‐ and time‐dependent manners. (A) Dose‐dependent effects of Fgf‐2 on *Npnt* mRNA expression. MC3T3‐E1 cells were treated with 0, 0.01, 0.1, 1, 10, or 100 ng·mL^−1^ of Fgf‐2 for 24 h. Values are shown as the mean ± SD of three samples as compared to the level in cells without treatment. **P < 0.01, relative to level in untreated cells (ANOVA). (B) Time‐dependent effects of Fgf‐2 on *Npnt* mRNA expression in MC3T3‐E1 cells. Cells were treated with 1 ng·mL^−1^ of Fgf‐2 for 6, 12, 24, or 48 h. Total cellular RNA was extracted, and then, mRNA levels for *Npnt* and *Gapdh* were examined using real‐time PCR. Values are shown as the mean ± SD of three samples as compared to the value at 0 h. *P < 0.05, **P < 0.01; relative to level in cells without treatment at each time point (Student's *t*‐test).

**Figure 3:**
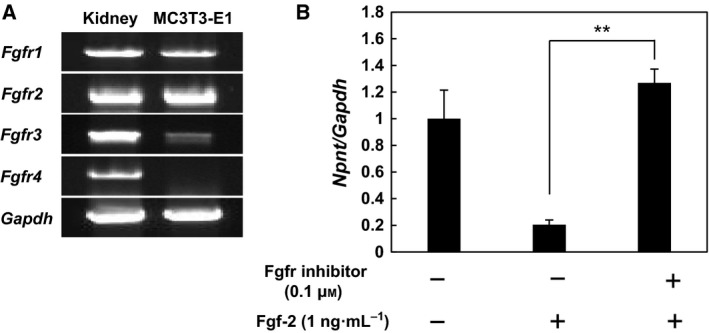
Expression of *Fgfrs* in MC3T3‐E1 cells and their involvement in expression of *Npnt*. (A) *Fgfr* expression in MC3T3‐E1 cells and kidney specimens. The expressions of *Fgfr1*,* Fgfr2*, and *Fgfr3* were examined using semiquantitative RT–PCR. (B) Inhibition of *Npnt* expression by Fgf‐2 was abrogated by the *Fgfr*s. Following pretreatment with 0.1 μm of an Fgfr inhibitor (BGJ398) for 1 h, MC3T3‐E1 cells were treated with 1 ng·mL^−1^ of Fgf‐2 and the Fgfr inhibitor for 24 h. Real‐time PCR was performed using cDNA derived from total cellular RNA from each sample to determine the expression levels of *Npnt* and *Gapdh* mRNAs. Results are shown as the mean ± SD of three samples as compared to the group without treatment. **P < 0.01 (ANOVA).

**Figure 4:**
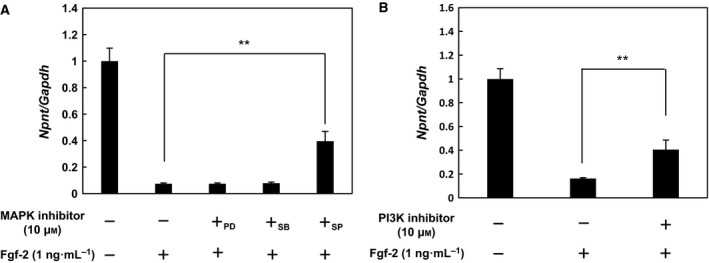
Treatment with MAPK and PI3K inhibitors following Fgf‐2 treatment. (A) Inhibition of *Npnt* expression by Fgf‐2 is regulated by JNK. Following pretreatment with MAPK inhibitors (+_PD_: 10 μm PD98059, +_SB_: 10 μm SB203580, +_SP_: 10 μm SP600125) for 1 h, and MC3T3‐E1 cells were treated for 24 h with 1 ng·mL^−1^ of Fgf‐2 and each MAPK inhibitor. Real‐time PCR was performed using cDNA derived from total cellular RNA obtained from each sample to determine the expression levels of *Npnt* and *Gapdh* mRNAs. Values are shown as the mean ± SD of three samples as compared to the group without treatment. **P < 0.01 (ANOVA). (B) Inhibition of *Npnt* expression by Fgf‐2 is regulated by PI3K. Following pretreatment with 10 μm of the PI3K inhibitor (LY294002) for 1 h, MC3T3‐E1 cells were treated for 24 h with 1 ng·mL^−1^ of Fgf‐2 and the PI3K inhibitor. Real‐time PCR was performed using cDNA derived from total cellular RNA from each sample to determine the expression levels of *Npnt* and *Gapdh* mRNAs. Values are shown as the mean ± SD of three samples as compared to the group without treatment. **P < 0.01 (ANOVA).

All results are expressed as the mean ± standard deviation (SD). For results shown in Figs 1A, 2A, 3B and 4A,B, statistical analysis was performed using one‐way ANOVA, while those shown in Figs 2B and S1 were analyzed using a two‐tailed Student's *t*‐test. A *P* value of < 0.05 or < 0.01 was considered to indicate statistical significance.

## Results

### Fgf‐2 strongly suppressed *Npnt* expression in Fgfs

We attempted to determine whether the expression of *Npnt* is regulated by members of the Fgf family, including Fgf‐1, Fgf‐2, Fgf‐8b, Fgf‐9, and Fgf‐23. MC3T3‐E1 cells were exposed to each of the investigated Fgfs for 24 h, and then, the level of *Npnt* mRNA was examined using real‐time PCR analysis (Fig. 1A). Fgf‐2 showed significant suppression of *Npnt* mRNA expression, while suppression by Fgf‐1 was also noted, although the level was not as great as seen with Fgf‐2. With 1 ng·mL^−1^ of Fgf‐2, the expression level of Npnt protein was also suppressed in MC3T3‐E1 cells after 24 h of incubation (Fig. 1B). Thus, we exposed MC3T3‐E1 cells to various doses of Fgf‐2 for 24 h and found that downregulation of *Npnt* expression occurred in a dose‐dependent manner, with the level reaching a plateau at ~ 1 ng·mL^−1^ (Fig. 2A). Finally, we investigated time‐dependent suppression by Fgf‐2 at a dose of 1 ng·mL^−1^ and detected a significant decrease in *Npnt* mRNA at 6 h after its addition to culture (Fig. 2B). The rate of *Npnt* expression by cells treated with Fgf‐2 as compared to untreated cells was decreased in a time‐dependent manner, with the level reaching a plateau at 24 h (data not shown).

### Expression of *Fgfr* genes in MC3T3‐E1 cells and their involvement in *Npnt* expression

The *Fgfr* (Fgf receptor) gene family is comprised of four members, *Fgfr1*‐*Fgfr4* 8. We found that each was well expressed in kidney specimens, while *Fgfr1‐3* were expressed in MC3T3‐E1 cells (Fig. 3A). After treating MC3T3‐E1 cells with BGJ398, an Fgfr inhibitor, real‐time PCR analysis was performed, which showed that 0.1 μm of BGJ398 blocked suppression of *Npnt* mRNA induced by 1 ng·mL^−1^ of Fgf‐2 and restored its expression (Fig. 3B).

### *Npnt* expression regulated by Fgf‐2 via JNK and PI3K pathways

Fgfr‐mediated signaling initiates activation of the mitogen‐activated protein kinase (MAPK) and PI3K 16. Downstream of MAPK and PI3K, Fgfr signaling has been shown to regulate several distinct MAPKs, including extracellular signal‐regulated kinase (ERK)1/2, p38, and JNK 17, 18, 19. To examine the molecular mechanism of *Npnt* mRNA downregulation by Fgf‐2, we first examined its relationship with the MAPK pathway. MC3T3‐E1 cells were treated with Fgf‐2 at 1 ng·mL^−1^, followed by 10 μm of PD98059 (MAPK‐ERK kinase inhibitor), SB203580 (p38 MAPK inhibitor), or SP600125 (JNK inhibitor). Of those, treatment with SP600125 inhibited the suppression of *Npnt* mRNA expression by Fgf‐2 (Fig 4A). To examine the PI3K pathway, MC3T3‐E1 cells were treated with Fgf‐2, then with 10 μm of LY294002 (PI3K inhibitor), which showed that downregulation of *Npnt* mRNA expression was inhibited by treatment with LY294002 (Fig. 4B).

## Discussion

**Figure 5:**
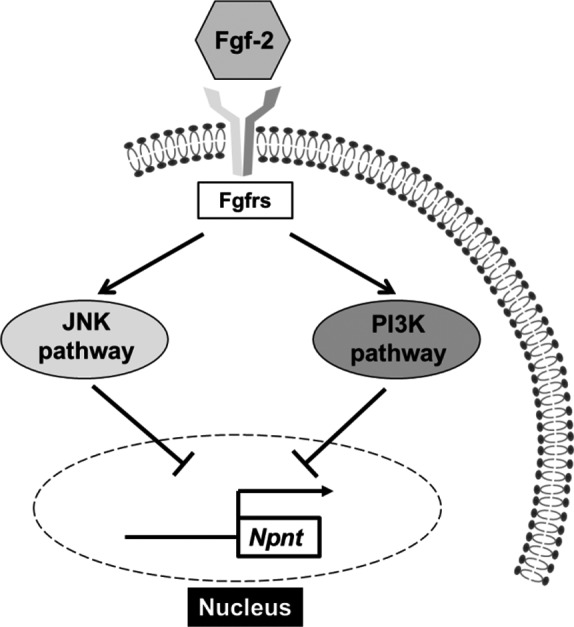
Fibroblast growth factor (FGF)‐2 downregulation of *Npnt* gene expression via JNK and PI3K pathways. Following activation by Fgf‐2 in MC3T3‐E1 cells, Fgfr is coupled to the intracellular signaling pathway, including the JNK and PI3K pathways, and then, *Npnt* expression is suppressed.

This study is the first to show that Fgf‐2 strongly inhibits *Npnt* mRNA expression in a manner related to the JNK and PI3K signaling pathways (Fig. 5). Npnt, which enhances osteoblast differentiation, is expressed in the basement membrane of developing teeth and extracellular matrix of developing jawbones 5. In addition, Linton *et al*. 20 showed that embryos lacking a functional *Npnt* gene frequently display kidney agenesis or hypoplasia, which could be traced to a delay in invasion of metanephric mesenchyme by the ureteric bud at an early stage of kidney development. It has been speculated that kidney disease causes disordered mineral metabolism, resulting in bone disease and ultimately fracture 21; thus, it is considered that Npnt is closely related to bone metabolism. The relationship of Fgf‐2 with skeleton development and bone metabolism has been reported in several studies. For example, overexpression of human FGF‐2 in mice (TgFGF‐2) results in dwarfism, with shortening and flattening of long bones and moderate macrocephaly 22, while its deletion has been shown to lead to decreased levels of bone mass, formation, and mineralization in mice 23, 24.

A previous study also demonstrated that Fgfs activates PI3K signaling 8; thus, we investigated whether *Npnt* mRNA expression suppressed by Fgf‐2 is also regulated via the PI3K pathway. Several different materials, such as Igf‐1 25 and ghrelin 26, have been reported to activate PI3K signaling, while the present results also suggest that these substances may regulate *Npnt* mRNA expression.

We used the osteoblast‐like MC3T3‐E1 cell line in the present study and also examined primary osteoblasts obtained from calvaria of 1‐day‐old mice. Fgf‐2 inhibited the expression of *Npnt* mRNA in primary osteoblasts (Fig. S1). It has also been reported that *Npnt* enhances osteoblast differentiation, while contrasting findings showed that FGF‐2 increases osteoblast differentiation and extracellular matrix mineralization *in vitro* 27, 28, 29. Similar contradictory results were shown in another study, which found that expression of a transcriptional co‐activator with a PDZ‐binding motif (Taz) was regulated by Fgf‐2 30. These results might help to explain the complex mechanisms of Fgfs.

## Conclusions

Fibroblast growth factor‐2 suppresses *Npnt* mRNA expression in MC3T3‐E1 cells in a dose‐ and time‐dependent manner by activation of the JNK and PI3K pathways. Our results suggest novel mechanisms related to *Npnt* gene expression.

## Author contributions

TK, AY, MI, YY, KS, NM, AS, TI, DC, HO, and RK involved in study concept. TK, AY, MI, YY, KS, and RK: collected the data; AY, MI, YY, KS, and RK: involved in formal analysis; AS, HO, and RK: acquired funding; TK: investigated the study; TK, AY, and RK: applied methodology for the study; AY and RK: administrated the project; AS, HO, and RK: collected the resources; AY and RK: supervised and validated the study; TK and AY: wrote the original manuscript; TK, AY, and RK: reviewed and edited the original manuscript.

## Supporting information


**Fig. S1**. Fgf‐2 inhibits expression of *Npnt* in primary osteoblasts. Real‐time PCR analysis was performed using cDNA from primary osteoblasts after treatment with 1 ng.mL^−1^ of Fgf‐2 for 24 hours. Values are shown as the mean ± SD of 3 samples as compared to the value without Fgf treatment. *p<0.05, **p<0.01; relative to level in cells without treatment (Student's *t*‐test).
