# Supplementary figures and images for: FGF‐2 suppresses expression of nephronectin via JNK and PI3K pathways
Source: FEBS Open Bio. 2018 Apr 19;8(5):836–42. doi: 10.1002/2211-5463.12421 (PMC5929927; doi:10.1002/2211-5463.12421)

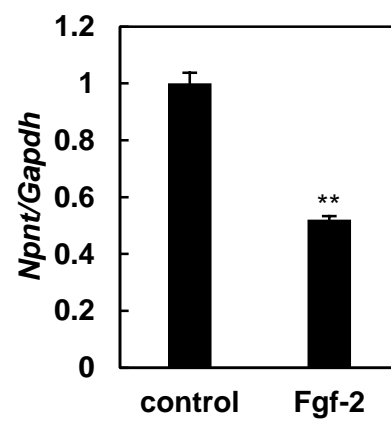

S Fig. 1. Kato T. *et al.*

Supplement: Supplementary file 1 — Fig. S1. Fgf‐2 inhibits expression of Npnt in primary osteoblasts. Real‐time PCR analysis was performed using cDNA from primary osteoblasts after treatment with 1 ng.mL−1 of Fgf‐2 for 24 hours. Values are shown as the mean ± SD of 3 samples as compared to the value without Fgf treatment. *p<0.05, **p<0.01; relative to level in cells without treatment (Student's t‐test). [file FEB4-8-836-s001.pdf]
